# Mechanistic Insight Into the Antifungal Effects of a Fatty Acid Derivative Against Drug-Resistant Fungal Infections

**DOI:** 10.3389/fmicb.2020.02116

**Published:** 2020-09-08

**Authors:** Anamika Bhattacharyya, Mau Sinha, Himanshi Singh, Ranjeet Singh Patel, Sumana Ghosh, Kabir Sardana, Shamik Ghosh, Shiladitya Sengupta

**Affiliations:** ^1^Vyome Therapeutics Ltd., New Delhi, India; ^2^Department of Dermatology, Atal Bihari Vajpayee Institute of Medical Sciences, Dr. Ram Manohar Lohia Hospital, New Delhi, India; ^3^India Innovation Research Center, New Delhi, India; ^4^Division of Engineering in Medicine, Department of Medicine, Brigham and Women’s Hospital, Harvard Medical School, Boston, MA, United States

**Keywords:** fatty acids, antifungals, dermatophytosis, candida, drug resistance

## Abstract

The prevalence of drug-resistant pathogenic fungi is a major global health challenge. There is an urgent need for novel drugs that can exert a potent antifungal activity and overcome resistance. Newly discovered anti-fungal properties of existing compounds can potentially offer a rapid solution to address this persistent threat. We rationalized that structures which disrupt the fungal cell membrane could address the above unmet need. As fatty acids underpin the formation and stability of cell membranes, we used computational simulations to evaluate the interactions between selected short chain fatty acids and a model cell membrane. Here, we report that caprylic acid could penetrate and perturb the membrane *in silico*. Based on the *in silico* findings, we identified a derivative of this fatty acid that disrupts fungal membranes as detected using steady-state fluorescence anisotropy. We show that this fatty acid derivative is potent against a variety of fungal pathogens like *Candida* and *Trichophyton*. We further demonstrated the ability of this fatty acid derivative to potentiate some azoles *in vitro* and enhance the efficacy of antifungal formulations *in vivo.* Our data suggests the emergence of a novel therapy for effective disease management and overcoming anti-fungal drug resistance.

## Introduction

The emergence of drug-resistant microbes is a major global challenge. While the term “drug-resistant microbes” is most commonly associated with antibiotic-resistant bacteria, the Centers for Disease Control and Prevention (CDC) has recently highlighted drug-resistant fungal infections as an emerging threat.^1^ The limited arsenal of antifungal agents and the paucity of novel antifungal agents in the pipeline exposes a serious vulnerability in our preparation to deal with this existing challenge, concerns about which were raised almost two decades ago (Sanglard and Odds, 2002). Pathogenic microbes appear to be evolving at a pace that is not matched with the long development process of new antibiotics or antifungals.^2^ There is an urgent need for novel molecules that can exert a potent antifungal activity or overcome resistance. One strategy to circumvent this crisis is to identify novel antifungal activities among compounds, such as fatty acids, that are already approved or considered as safe by the regulatory agencies such as the United States Federal Drug Administration (USFDA). Previous studies have reported both pro-growth and inhibitory activity of fatty acids against microbes (Nieman, 1954; Desbois and Smith, 2010; Pohl et al., 2011). Fatty acids exert their anti-microbial actions by targeting different cellular functions including protein synthesis, fatty acid metabolism and even topoisomerase activity (Pohl et al., 2011). More recently, Jadhav et al. (2017), demonstrated that capric and caprylic acids inhibit processes involved in *Candida albicans* virulence like morphogenesis, adhesion and biofilm formation. However, their primary mode of antifungal action is through membrane perturbations in the target organism (Pohl et al., 2011). Interactions of fatty acids with membranes and the subsequent disruption of the bilayer are governed by several factors including the structural properties of the fatty acid (chain length, degree, position and orientation of unsaturation, etc.) as well as those of the target membrane (Desbois and Smith, 2010). Longer chain length increases the hydrophobicity of the fatty acid and its antimicrobial properties but may reduce solubility in an aqueous medium and prevent interactions with the acyl chains of the membrane phospholipids. A scientific understanding of these behaviors could enable the repositioning of fatty acids and/or their derivatives as novel treatments or potentiators of known antifungals against drug-resistant fungi.

We have used an array of *in silico*, *in vitro*, and *in vivo* approaches to compare the effect of two fatty acids of distinct carbon lengths, i.e., lauric acid (C12) and caprylic acid (C8), on a model membrane and on the growth of drug resistant fungal pathogens. Fatty acids with greater than 12 carbon atoms are key components of cell membranes (Guidotti, 1972; Khuller et al., 1981; Stahl and Klug, 1996). We rationalized that fatty acids with shorter than 12 carbon atoms could potentially exert an antifungal effect by perturbing the cell membrane. Indeed, *in silico* molecular dynamics (MD) simulation using the Martini coarse grain (CG) model (Marrink et al., 2007; Marrink and Tieleman, 2013), to study the interactions of the two fatty acids with a model lipid bilayer membrane, revealed that caprylic acid (CAP) displayed an inherent propensity to interact with and penetrate the model membrane while lauric acid (LRA) formed self-clusters that barely interacted with the bilayer. Our simulation data provided a molecular basis for the mechanisms by which CAP may exert immediate and potent membrane penetrative actions. Interestingly, *in vitro* analysis of fungal membrane fluidity upon treatment with CAP suggested large scale decrease in the bilayer order which was not seen upon exposure to LRA. This observation of fungal membrane perturbation by CAP aligns with existing literature reports of membrane damage caused by CAP in *C. albicans* and *S. cerevisiae* (Liu et al., 2013; Bae and Rhee, 2019). Further, our *in vitro* time kill assays and electron microscopy data show CAP and its ester derivative, propylene glycol monocaprylate (PGMC), to have potent membrane disruptive actions against various fungal pathogens. We also demonstrate *in vitro* synergistic action of PGMC and azole antifungals with improved *in vitro* efficacy of antifungal formulations containing PGMC against *C. albicans* and *Trichophyton* spp. To validate our observations, two distinct superficial skin infection studies were performed in clinically relevant and experimentally robust animal model systems of cutaneous candidiasis and tinea wherein azole formulations containing PGMC were compared to formulations currently in clinical use. Our results indicate the potential advantages of combining azoles (and maybe other classes of antifungals) with CAP/PGMC as an effective treatment strategy for resistant and difficult-to-treat fungal infections.

## Materials and Methods

### Fungal Strains

*M. furfur* (MTCC 1374) and *C. albicans* (MTCC 227) were obtained from MTCC (IMTECH, Chandigarh, India). *T. rubrum* (ATCC 28188) was procured from Himedia. *C. albicans* (ATCC 22972) and *T. mentagrophytes* (ATCC 24953) were procured from LGC Promochem (Bangalore, India). *Trichophyton mentagrophytes var interdigitale* (KA01) was isolated from a patient sample as part of an epidemiological study with Dr. K Sardana, Ram Manohar Lohia Hospital, Delhi (manuscript under preparation). The sample collection protocol was endorsed through an approval (190(9/2017)/IEC/PGIMER/RMLH)1316/17 Dt. Aug 14, 2017 by Institutional ethics committee, PGIMER, Dr. Ram Manohar Lohia Hospital, New Delhi, India.

### Animals

Animal studies were performed at TheraIndx Lifesciences Pvt. Ltd. (Bangalore, India) following all ethical practices as laid down in the guidelines for animal care entitled “CPCSEA Guidelines for Laboratory Animal Facility” [as per Govt. of India, Committee for the Purpose of Control and Supervision of Experiments on Animals (CPCSEA), Registration Number: 1852/PO/Rc/S/16/CPCSEA]. BALB/c mice were procured from Vivo Biotech Ltd. (Hyderabad, India).

### Computational Methods

#### Adaptation of the MARTINI CG Model

The MARTINI Coarse Grain model (Marrink et al., 2007; Marrink and Tieleman, 2013) was used to model the cell membrane constructed from a pre-assembled lipid bilayer containing 1-palmitoyl-2-oleoylsn-glycero-3-phosphocholine (POPC) lipid molecules. Bonded and non-bonded parameters for POPC were adapted from the standard MARTINI force field for lipids (Marrink et al., 2004). MARTINI force field bead types for the POPC molecule are depicted in Supplementary Figure 1. In the MARTINI CG force field, lipid head groups are depicted with two charged beads followed by two intermediate polarity beads. The hydrophobic lipid tails are modeled with apolar beads. CAP and LRA molecules are modeled with MARTINI force field parameters for surfactants (Marrink et al., 2004, 2007). The hydrophilic heads of the CAP and LRA molecules were modeled with the polar, uncharged MARTINI bead type (Supplementary Figure 1). Hydrophobic CAP and LRA tails are represented by apolar MARTINI CG beads. In the MARTINI force field, water molecules are represented by a single bead that matches four atomistic water molecules.

#### Simulation Set Up

Simulations were performed using Gromacs 4.5.5 (Hess et al., 2008; Pronk et al., 2013). The temperature of the system was maintained at 300 K using a V-rescale thermostat. Initial 50 ns equilibration run was performed maintaining the pressure at 1 bar using Berendsen barostat (Berendsen et al., 1984) with semi-isotropic pressure coupling. This was followed by final simulation using the Parrinello-Rahman barostat (Parrinello and Rahman, 1981) with semi-isotropic pressure coupling. In the starting configuration of the simulation, CAP or LRA molecules were randomly placed in water and equilibrated. The system containing 1000 CAP molecules were simulated with 1440 POPC molecules and 66653 CG Martini water beads, and the systems with 522, 272, and 122 CAP molecules with 1440 POPC molecules and 66584 CG Martini water beads. Further, the LRA containing system was simulated with 1000 LRA molecules, 1440 POPC molecules, and varying number of CG Martini water beads. The number of Martini CG water beads were increased to 69,500 such that the ratio of total number water beads and the sum of POPC and LRA beads was same as in the 1000 CAP containing system. The final simulation was of 1000 ns for CAP molecules and 1500 ns for LRA molecules.

For the CAP containing system, mean square displacement (MSD) for the POPC molecules within the lipid bilayer were computed at three time patches (each 5 ns long) and 50 ns apart near the time range of transition in the non-bonded potential energy using the g_msd tool in Gromacs. Diffusion constants were calculated from Einstein’s relation by least square fitting a straight line to the mean MSD plot discarding 10% points at the beginning and end. The trend in diffusion constants for the systems containing 1000, 522, 272, and 122 CAP molecules was obtained by calculating the diffusion constant from the mean MSD plot computed near the time range of transition in the non-bonded potential energy. For the LRA containing system, MSDs were calculated at three distinct time spans (each 5 ns long) and 50 ns apart from last 100 ns of the trajectory as the transition in the non-bonded potential energy did not occur in the representative LRA system.

Bond order parameters were calculated using the do-order-multi.py script^3^. To compute the bond order parameter, first the average bond order parameter was calculated from the same three time spans for which MSDs were calculated (each time span 5 ns long, and 50 ns apart near the time range of transition in the non-bonded potential energy). The plots for the systems containing 1000, 522, 272, and 122 CAP molecules were generated from the average bond order parameter calculated at the same three-time spans for which MSDs were calculated. The mean bond order parameter for the representative LRA system was obtained from the average bond order parameter calculated from the same three-time spans from last 100 ns of the trajectories for which MSDs were calculated.

### *In vitro* Time Kill Assay in *Malassezia furfur*

*M. furfur* (MTCC 1374) inoculums (1 × 10^7^ cells) were prepared in sterile Sabouraud Dextrose Broth (SDB). Time-kill assays were performed as described (Rukayadi et al., 2006) with slight modifications. Fatty acids or their monoesters were tested at a final working concentration of 0.02%. A vehicle control (DMSO) was included for each experiment. The samples were incubated at 32 ± 2°C for 3 h with intermittent sampling. For colony forming unit (CFU) counts, cell samples at various time points were serially diluted in SDB medium containing 0.1% Triton-X-100 and spread on Sabouraud Dextrose Agar (SDA) plates containing 2% olive oil. The plates were incubated at 32 ± 2°C for 72 h.

### *In vitro* Dose Response Assay in *Malassezia furfur*

*M. furfur* (MTCC 1374) cell suspensions (1 × 10^7^ cells per sample) were prepared in SDB supplemented with 2% olive oil. Fatty acids or their propylene glycol monoesters were tested at final concentrations of 0.02, 0.05, 0.1, or 0.2%. A vehicle control contained 2% DMSO. All samples were rotated end-over-end (20 rpm) for 30 min, to allow uniform exposure to the test agents. At the end of the treatment samples were diluted in SDB and spread on SDA plates containing 2% olive oil. The plates were incubated for 72 h at (32 ± 2)°C for final CFU counts.

### SEM and TEM Analysis

*M. furfur* cells grown on Leeming Notman agar (LNA) plates for 2–3 days were suspended in SDB and vortexed to disrupt cell clumps. Test agents (fatty acids or their esters) were added to the cell suspension at a final concentration of 0.2%. The treated and control samples were rotated end-over-end (20 rpm) at room temperature for 30 min and washed with phosphate buffered saline (PBS) three times. The final cell pellet was suspended in 1 mL PBS and processed for analysis by scanning (EVO18 Zeiss) and transmission (Tecnai, G 20 [FEI]) electron microscopy. *C. albicans* strain ATCC 22972 was grown on SDA plates. Cells in SDB (1 × 10^6^) were used for treatment with luliconazole at its MIC value (4 μg/mL), or PGMC at 0.2% or a combination of luliconazole and PGMC for 3 h at 37°C (rotated end-over-end at 20 rpm). The treated samples were washed three times with 0.05 M sodium cacodylate buffer (pH 7.4) and fixed with 4% paraformaldehyde and 2.5% glutaraldehyde solution for 16–18 h at 4°C, as described previously (Hanschke and Schauer, 1996). Post fixation, cells were washed twice with 0.05 M sodium cacodylate buffer (pH 7.4) and resuspended in fresh 0.05 M sodium cacodylate buffer for analysis by scanning electron microscopy.

### Determination of Membrane Fluidity Using Fluorescence Anisotropy

Changes in membrane fluidity due to CAP/LRA/PGMC treatment in fungal cells were measured using fluorescence polarization of the hydrophobic probe 1,6-diphenyl-1,3,5-hexatriene (DPH). Fungal cells were treated with different concentrations of CAP and LRA for four different time points (5, 10, 20, and 30 min) at 32°C. The cells were then incubated with DPH (2 μM) at room temperature for 30 min as described previously (Kohli et al., 2002). Fluorescence anisotropy values were monitored using a SpectraMax 5 Multi-Mode microplate reader (Molecular Devices) at room temperature using excitation and emission wavelengths at 360 and 460 nm, respectively. The calculated fluorescence intensities were corrected for background fluorescence and light scattering from unlabeled cells.

### Preparation of Antifungal Cream Formulations Using PGMC

Topical antifungal cream formulations were prepared in-house using oil-in-water (O/W) emulsion technology (Nastiti et al., 2017). Briefly, the active pharmaceutical ingredient (clotrimazole or luliconazole) was accurately weighed (calculated to achieve 1% in final formulation) and dissolved in a mixture of lipid and surfactant containing solution at 50°C to obtain a transparent phase A solution that includes PGMC as one of the lipid components. The phase A solution was then added into 0.1% Carbopol (gelling agent) containing phase B solution under continuous stirring condition. The resultant mixture was then allowed to cool to room temperature followed by addition of preservative, anti-oxidant and pH modifiers to yield final cream-based formulation. Formulations without the active pharmaceutical ingredient were considered as placebo formulations and contained PGMC. Detailed physico-chemical characterizations were performed with the final formulations. In addition, the drug content in the formulations were measured by HPLC at zero time and 3-month post storage at accelerated stability condition (40°C and 75% relative humidity). For both clotrimazole and luliconazole formulations, the drug content was found to be 1 ± 0.1% which allowed to predict stability at room temperature for a year.

### Zone of Inhibition Assays Using Antifungal Formulations

*In vitro* fungal killing efficacy of clotrimazole (1%) or luliconazole (1%) formulations were studied using zone of inhibition (ZOI) assays. Each formulation was diluted in sterile water (1:10 for clotrimazole formulations and 1:100 for luliconazole formulations). Sterile discs for ZOI assays were placed in the center of SDA plates inoculated with either *T. rubrum* (ATCC 28188) or *C. albicans* (MTCC 227). Ten microliter of the diluted formulations were loaded onto each disc and the plates were incubated for 5 days at 37°C for *Trichophyton* or 24 h at 32°C for *Candida*. The zones of inhibition were measured at the end of the incubation period. The assays were performed in triplicate for each formulation.

### Checkerboard Assays to Assess Combination Effects of Two Agents

Potentiation of the activity of antimycotic agents using PGMC was tested using the checkerboard method (Hsieh et al., 1993) by serial dilution of each agent in SDB. Inoculum of relevant fungal strains (*Trichophyton* or *Candida*) were added to the wells with various drug combinations and observed for growth inhibition at the end of the incubation period set by the protocol. The fractional inhibitory concentration (FIC) value for a drug in a particular well of the checkerboard layout, was calculated by dividing the drug concentration in that well by the established MIC value of the drug against the test organism (Hsieh et al., 1993). FIC values for both test agents, in a particular well were used to determine the FIC index (sum of the FICs of each drug in that well). Combinations that gave FIC indices < 0.5 were designated “synergistic” (Meletiadis et al., 2010; Zhang et al., 2015).

### *In vitro* Time Kill Assays Using Antifungal Formulations in *C. albicans*

Time kill assays in *C. albicans* (MTCC 227) were performed using the method described earlier (Klepser et al., 1997) with some modifications. *C. albicans* inoculum (1 × 10^6^ cells/mL) was prepared in SDB. The test antifungal formulations were diluted 10 times in sterile water. 0.1 mL diluted formulation was added to 0.9 mL of the culture suspension for treated samples. For the broth control, 0.1 mL sterile water was added to 0.9 mL culture suspension. The samples were incubated at 32°C for 24 h on a tube rotator (20 rpm) with sampling at pre-determined time points (0, 1, 6 and 24 h). For CFU counts, 0.5 mL culture was removed from each sample, diluted in SDB, and spread on SDA plates. The plates were incubated at 32°C for 24 h before enumerating the colonies.

### *In vivo* Efficacy Studies in Fungal Skin Infection Models

#### Candida Skin Infection Efficacy Study

BALB/c mice were dosed with intra-peritoneal injection of cyclophosphamide (150 mg/kg) on days-4 and -1 prior to infection with *C. albicans* (MTCC 227). On the day of infection (day 0), the dorsal skin of mice under anaesthesia [using ketamine (70 mg/kg) and xylazine (20 mg/kg)] were shaved with electric clippers and lightly abraded (1 × 1 cm). A 40 μL suspension of *C. albicans* (∼4 × 10^7^ CFU/animal) was applied to the abraded skin. Twice daily treatments (12 h apart) were initiated 24 h post infection with the topical application (750 mg formulation/kg/dose) of clotrimazole (1%) formulation containing PGMC or marketed formulation of 1% clotrimazole, each twice daily (12 h between each dose). The reference marketed formulation of 1% clotrimazole (Glenmark Pharmaceuticals Ltd., India) was commercially available for clinical use. Animals were sacrificed at 4 timepoints (24, 36, and 48 h post infection) and samples were collected from the infected skin, using sterile cotton swabs. The swabs were extracted with 1.0 mL of sterile SDB. The samples were serially diluted and 50 μL of each dilution was plated on sterile SDA plates and incubated at 25°C for 48 h. The skin lesions were visually examined daily throughout the experiment to determine the severity and recovery of lesion.

#### Tinea Skin Infection Efficacy Study

Tinea infection was induced in the dorsal skin of BALB/c mouse using the strain *Trichophyton mentagrophytes* (ATCC 24953). Animals were rendered neutropenic as described in previous section. On day 0, a *T. mentagrophytes* culture (∼1.5 × 10^8^ CFU/mL of live spores), was applied to the abraded skin of mice anaesthetized using 3–5% isoflurane. Topical treatment (750 mg formulation/kg/dose) was initiated on the 5th day post-infection by application of luliconazole (1%) formulation containing PGMC or the marketed formulation of 1% luliconazole, twice daily (12 h between each dose) and continued until day 14 post infection. The reference marketed formulation of 1% luliconazole (Sun Pharmaceutical Ind. Ltd., India) was commercially available for clinical use. Skin lesions were scored on days 5, 10, 15, and 21 post infection and graded as follows: 0 – absence of lesion; 1 – appearance of erythema at infected site or new hair growth on the bald exposed area; 2 – moderate erythema spreading over entire infected site; 3 – intense erythema with abrasions, swelling and scaling; and 4 – severely erythematous lesion with crusting spreading over the entire exposed area. The average lesion scores were calculated for each group to determine the severity and recovery of lesion till day 21 (Koga et al., 2012). Histopathology of the fungal infected skin tissue was done after harvesting the dorsal skin followed by fixation in 10% neutral buffered formalin. Skin was processed by paraffin block embedding, sectioned and stained with hematoxylin and eosin. The tissue sections were examined microscopically for inflammation, fungal cells, epidermal hyperplasia, and necrosis and scored in a tabulated form.

### Statistical Analysis

All the results are expressed as the means ± SD for the number of separate experiments indicated in each case (*n* ≥ 3). Student’s *t*-test was used for comparison between two groups.

## Results

### Distinct Modes of Interaction Between Model Membrane and Selected Fatty Acids

**FIGURE 1:**
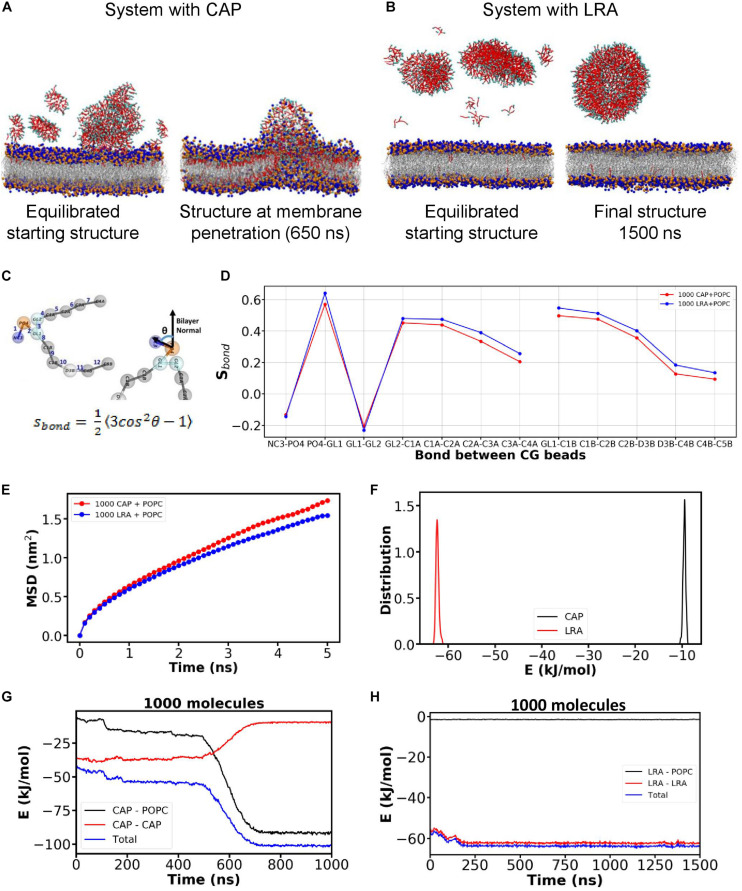
Molecular simulations show the interactions of CAP and LRA on a POPC bilayer. Snapshots from simulations of POPC bilayer systems containing **(A)** CAP, and **(B)** LRA. Head group of phospholipid molecules are depicted using blue and orange beads, phospholipid tails are represented with gray sticks. Hydrophobic tails for both CAP and LRA are represented with red beads and their hydrophilic head is represented as cyan beads. **(C)** Schematic representation depicting the bond between CG beads and formula for calculating the bond order parameter (S_bond_). **(D)** Changes in the S_bond_ value across different bonds for POPC-CAP and POPC-LRA systems depicting lipid tail ordering for both systems. **(E)** Mean square displacement (MSD) of lipid molecules in the bilayers of POPC-CAP and POPC-LRA systems indicate differences in membrane fluidity of the two bilayer systems. **(F)** The total non-bonded potential energy of interaction among LRA molecules versus that of CAP molecules computed as sum of Lennard Jones potential to understand which interactions are favorable. Non-bonded potential energy of interaction between different components of the system as a function of time for **(G)** the CAP-POPC system and **(H)** the LRA-POPC system during the simulation.

We performed coarse grain (CG) molecular dynamics simulation of CAP and LRA with a model lipidic membrane (refer Supplementary Figure 1) using the MARTINI forcefield for lipids (Marrink et al., 2007; Marrink and Tieleman, 2013). To model the cell membrane, we constructed a lipid bilayer using standard POPC lipids. The starting system for the simulation had CAP or LRA molecules randomly placed in the water layers on both sides of the lipid bilayer. Starting with random configuration of CAP or LRA in the water layer on top of the bilayer, the system was first equilibrated by performing simulation under NPT (constant particle number, pressure, and temperature) ensemble with semi-isotropic pressure coupling. The equilibrated starting structures used to perform the final simulations are depicted in Figures 1A,B (left). Starting from the equilibrated system, the CAP simulation was run for 1000 ns. The initially dispersed CAP molecules (Figure 1A; left) were found to self-aggregate in water and then interact with the bilayer, and finally penetrated the bilayer within 1000 ns. On the other hand, the LRA molecules formed and remained as an aggregate in the water layer for the entire duration of 1500 ns in the LRA system (Figure 1B). Data from single representative simulation trajectories for CAP and LRA are presented. In the representative trajectory for CAP, after about 500 ns, the CAP aggregates started penetrating the bilayer, forcing CAP molecules to enter the bilayer. During the entry of CAP molecules into the lipid bilayer, in the snapshot at 650 ns in the representative trajectory (Figure 1A; right), the hydrophilic head groups of the lipid molecules appeared to have folded into the hydrophobic region of the bilayer (Figure 1A, right). The penetration of CAP molecules into the bilayer is captured in the movie (Supplementary Information Movie_CAP-1000). In contrast, the LRA molecules also self-aggregated but formed large clusters and were unable to penetrate the bilayer (Figure 1B) in the representative system. This is captured in the movie for LRA containing system (Supplementary Information Movie_LRA-1000).

### CAP and LRA Differentially Affect Membrane Order

To understand the effect of fatty acid on the packing of lipid molecules, the bond order parameter or S_bond_ (Marrink et al., 2004) for each bond in the phospholipid model was calculated (Figure 1D). The equation for computing the bond order parameter is shown in Figure 1C (bottom) where, θ is the angle between vector along any CG bond (harmonic bond potential between two connected CG beads as defined in the MARTINI CG model) and bilayer normal (represented schematically in Figure 1C; top). A plot of mean S_bond_ as a function of bond between CG beads is depicted in Figure 1D. The mean S_bond_ for lipid CG bonds is lower in the CAP containing system (Figure 1D; red curve) compared to the LRA containing system (Figure 1D; blue curve). To understand the differences in the dynamics i.e., the membrane fluidity of bilayers in the CAP and LRA containing systems, MSD of the phospholipid molecules in the bilayers of each system were calculated (Figure 1E). Mean MSD plots computed from MSDs obtained at three time patches are shown for CAP containing system (1000 CAP molecules), and LRA containing system (1000 LRA molecules) in Figure 1E. Compared to the LRA containing (blue curve) system, the MSD of phospholipid molecules in the system containing CAP molecules is higher (red curve).

We investigated the distinct membrane penetration behavior of CAP molecules unlike LRA. For this, the total non-bonded potential energy of interaction among the different constituents of the system was computed as the sum of Lennard-Jones (LJ) potentials. The distribution of the intermolecular non-bonded energy among the fatty acid molecules from the final 100 ns of the trajectory is depicted in Figure 1F. This suggests stronger inter-molecular interaction between the aggregated LRA molecules compared to that between CAP molecules. Finally, the fatty acid-fatty acid and fatty acid-phospholipid energy components as a function of time were analyzed (Figures 1G,H). For the CAP containing system, the CAP-phospholipid interaction energy became more favorable compared to CAP-CAP interaction energy over time. The crossover of energies occurred near 600 ns when the CAP clusters started penetrating the bilayer (Figure 1G). This region of transition, where CAP-CAP and CAP-phospholipid interaction energy plots cross each other, corresponds to the intermediate state where phospholipid molecules moved on top of the CAP aggregate, giving rise to a bell-shaped intermediate. Once the CAP molecules completely penetrate the phospholipid bilayer, their interaction energy with phospholipid molecules reached the minima. However, for the LRA containing system, over the whole simulation time, LRA-LRA interaction energy remained more favorable compared to LRA-phospholipid interaction energy (Figure 1H). As a result, LRA molecules form self-clusters and did not penetrate the phospholipid bilayer.

### Effect of Different CAP Concentrations on the Phospholipid Bilayer

**FIGURE 2:**
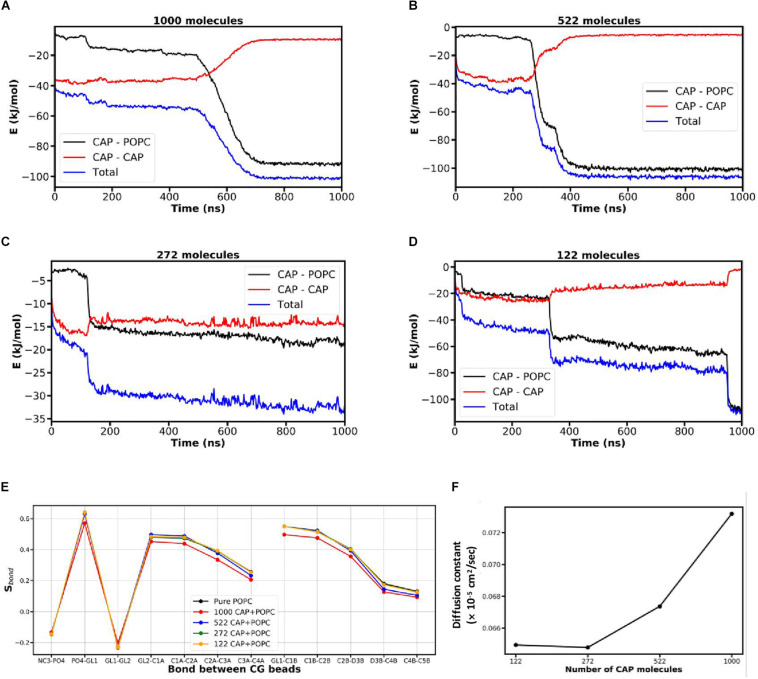
Effect of different concentrations of CAP on the POPC bilayer. **(A–D)** Transitions in the CAP-CAP and CAP-POPC components representing the time range at which CAP molecules penetrate the POPC bilayer. **(E)** Lipid tail ordering and **(F)** diffusion constant for the different concentrations of CAP computed using the non-bonded potential energy of interaction near the transition states due to penetration of CAP.

Since bilayer penetration was observed for the CAP-containing system, we next investigated the effect of varying concentrations of CAP molecules on the bilayer (Figure 2). Four different CAP amounts (122, 272, 522, and 1000 CAP molecules) were used in these simulations. Simulations were performed for 1000 ns for each CAP amount (movies in Supplementary Information). The simulation with 1000 CAP molecules (Movie_CAP-1000 in Supplementary Information) shows that starting from the initial equilibrated structure, the CAP molecules begin to self-aggregate and then interacted with the bilayer. The bilayer also started interacting with the nearby CAP aggregates as the phospholipid molecules climbed the surface of the CAP aggregates. In the movie (Supplementary Information Movie_CAP-1000), the blue and orange spheres representing the phospholipid head groups, slowly move up on the CAP aggregate, giving rise to the bell shaped intermediate. This enables the CAP molecules to penetrate the bilayer. In the 1000 CAP containing system at 650 ns (Supplementary Information Movie_CAP-1000 and Figure 2A), the hydrophilic head groups of the phospholipid molecules had folded inside the hydrophobic region of the bilayer. A similar mode of penetration by CAP molecules into the bilayer was found in the simulation containing 522 CAP molecules (Figure 2B and Movie_CAP-522 in Supplementary Information). However, at this CAP concentration, the lipid head groups of the membrane did not fold inside the hydrophobic region of the bilayer during the penetration of CAP molecules. This indicates that 522 CAP molecules may be insufficient to cause large scale perturbations in the bilayer as observed in the 1000 CAP containing system. For the simulation containing 272 CAP molecules, most of the CAP molecules remained aggregated in water after 1000 ns (Figure 2C and Movie_CAP-272 in Supplementary Information). In the simulation with 122 CAP molecules, CAP molecules entered the membrane without causing folding of the lipid head groups into the hydrophobic region of the bilayer (Figure 2D). To understand the effects of different quantities of CAP molecules on the packing of lipid molecules in the bilayer, we calculated the mean bond order parameter (S_bond_) for each bond in these bilayer systems near the time range of penetration of CAP molecules in the bilayer (Figure 2E). The 1000 CAP-containing system had the lowest S_bond_ among the simulations with different CAP concentrations which aligns with bilayer perturbation at this concentration.

To understand the effect of different CAP concentrations on the dynamics of phospholipid molecules in the bilayer, the diffusion constant of the phospholipid molecules in the bilayer was calculated with different quantities of CAP in the simulation system (Figure 2F) near the time range of penetration of CAP molecules in the bilayer represented by transition in the non-bonded potential energy plots (Figures 2A–D). The diffusion constant for each concentration of CAP molecules was calculated from least square fitting a straight line to the mean MSD plot computed near the transition of the non-bonded interaction energy representing the time range when CAP molecules penetrated the lipid bilayer. For lower CAP amounts (122 and 272 molecules), the diffusion constant of phospholipid molecules was relatively low. The diffusion constant gradually increased between 272 and 522 CAP molecules and then a steep increase in the presence of 1000 CAP molecules was observed. For simulations with 522/1000 CAP molecules, the intermediate structure in which POPC molecules move on the CAP aggregate (Movie_CAP-522, and Movie_CAP-1000 in Supplementary Information) contributes to the increased diffusion constant. With 1000 CAP molecules, the folding of lipid head groups inside the hydrophobic region in addition to the lipid molecules climbing on the CAP aggregates possibly resulted in the highest diffusion constant, suggesting perturbation of the bilayer (Figure 2F). As the lipid head groups do not fold into the hydrophobic region due to CAP penetration in the 522 CAP containing system, its diffusion constant is lower than the system containing 1000 CAP molecules.

### CAP but Not LRA Causes Membrane Perturbations in Fungal Cells *in vitro*

**FIGURE 3:**
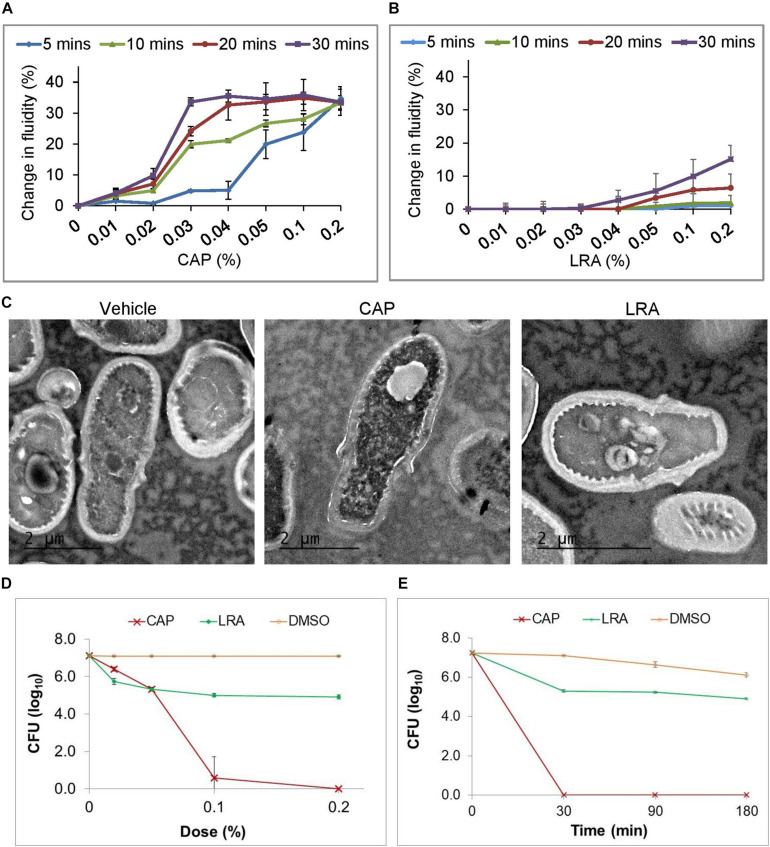
*In vitro* antifungal activities of CAP and LRA. Changes in membrane fluidity of DPH labeled *M. furfur* cells treated for 4 different durations of exposure to increasing concentrations of **(A)** CAP and **(B)** LRA (*n* = 3 for each group, each time point). **(C)** TEM images of vehicle-treated (left), CAP treated (center) and LRA treated (right) cells. **(D)** Effect of increasing dose of CAP or LRA on *M. furfur*. Cells in broth were treated with a fatty acid at the indicated concentrations (0.02, 0.05, 0.1, and 0.2%) for 30 min. Colony forming units (CFUs) were counted 48 h after plating and plotted as survival. **(E)** Effect of increasing duration of exposure of *M. furfur* to CAP or LRA tested at a dose of 0.2%. Cells were treated with CAP or LRA in broth and samples were plated at the indicated time points (30, 90, and 180 min). CFUs were counted 48 h after plating and plotted as survival. Data represent the average of at least three biological replicates with error bars indicating standard deviation.

Steady-state fluorescence anisotropy of 1,6-diphenyl- 1,3,5-hexatriene (DPH) labeled *Malassezia furfur* (MTCC 1374) cells was used to evaluate the membrane order of the cells treated with fatty acid. Membrane fluidity is inversely related to the fluorescence anisotropy parameter (Shinitzky and Barenholz, 1978). Addition of CAP caused a drastic change in fluidity in the fungal membranes in a time and dose dependent manner (Figure 3A). A 20% change in fluidity was apparent when the cells were treated with 0.03% CAP for 10 min and this effect peaked at about 32% after 30 min. Higher concentrations of CAP (up to 0.5%) increased membrane fluidity more rapidly but gave the same level of membrane fluidity after 30 min as 0.03% CAP. In contrast, the fungal cell membranes were less sensitive to treatment with LRA, showing only a marginal (between 6 and 18%) increase in membrane fluidity when the concentration of LRA was increased to 0.2% and treatment time to 30 min, suggesting a more modest perturbation of the fungal membrane due to the fatty acid treatment (Figure 3B).

### CAP Disrupts Membrane Structures in *M. furfur*

We visualized the morphological effects of short duration exposure of *M. furfur* cells to CAP or LRA treatment using transmission electron microscopy (TEM). A 30 min treatment resulted in morphological differences that clearly distinguished between vehicle-treated (Figure 3C, left), 0.2% CAP-treated (Figure 3C, center) and 0.2% LRA-treated (Figure 3C, right) cells. While the vehicle-treated control cells and LRA treated cells show clearly demarcated intra-cellular organization and intact organelles, CAP-treated cells had a disarrayed granular cytoplasm and an absence of membrane-bound organelles. In contrast to CAP, LRA had no effect on the membranes of *M. furfur*.

### Variable Growth Inhibitory Effects of Different Medium Chain Fatty Acids on *M. furfur*

The dose dependence of CAP and LRA on the growth of *M. furfur* was tested in liquid culture (Figure 3D). CAP strongly inhibited growth while LRA only partially inhibited growth at all doses tested. Figure 3E shows that 0.2% CAP or LRA induced cell death to their fullest extent within about 30 min of incubation.

### An Ester of Caprylic Acid Displays Antifungal Properties Through Membrane Disruptive Action

**FIGURE 4:**
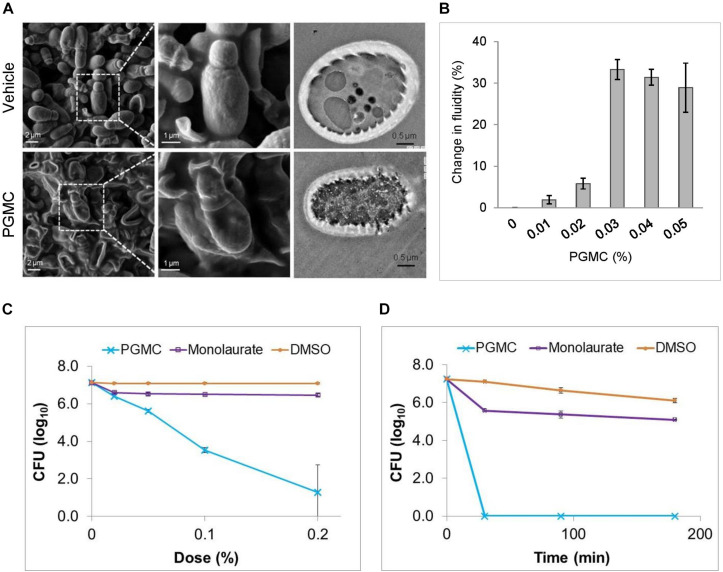
PGMC and CAP show similar antifungal activity. **(A)** SEM images of vehicle-treated *M. furfur* cells (left, upper panel) and those of PGMC treated (left, lower panel) cells. Images on the middle panels are magnifications of a group of cells demarcated by the squares from the field shown in the left panels of SEM images. TEM image of vehicle-treated *M. furfur* cell (right, upper panel) and that of a PGMC (0.2%) treated cell (right, lower panel). **(B)** Changes in membrane fluidity of DPH labeled *M. furfur* cells exposed to increasing concentrations of PGMC for 30 min (*n* = 3). **(C)** Effect of increasing dose of PGMC or a monoester of LRA (monolaurate) on *M. furfur*. Cells in broth were treated with the fatty acid monoesters at the indicated concentrations (0.02, 0.05, 0.1, and 0.2%) for 30 min. CFUs were counted 48 h after plating. **(D)** Effect of increasing duration of exposure of *M. furfur* to PGMC or monolaurate tested at a dose of 0.2%. Cells were treated with caprylic acid or its monoester in broth and samples were plated at the indicated time points (30, 90, and 180 min). CFUs were enumerated 48 h after plating. Data represent the average of at least three biological replicates with error bars indicating standard deviation.

The CAP ester, PGMC, exerted similar antifungal effects as CAP. A 30 min treatment of *M. furfur* cells with 0.2% PGMC resulted in morphological changes detected by SEM. Figure 4A shows vehicle-treated cells have a morphology consistent with normal turgor (top; left and center) while PGMC-treated cells have collapsed (bottom; left and center). The TEM images on the right (Figure 4A) show a vehicle treated cell with intact membranes (top) and a PGMC treated cell with disintegrated membranes (bottom). To test whether the membrane damaging effects of PGMC might be applicable across fungal species, we performed SEM analysis of *Candida albicans* after a 3 h treatment with vehicle, PGMC, luliconazole or a combination of PGMC and luliconazole (refer Supplementary Figure 2). PGMC treatment caused the *C. albicans* cells to shrink, as observed for *Malassezia*. The membrane perturbation effect of PGMC was confirmed by measuring changes in membrane fluidity using fluorescence anisotropy of DPH labeled cells (Figure 4B). Similar to CAP, PGMC concentrations of 0.03–0.05% gave a comparable (30%) increase in membrane fluidity in cells treated for 30 min. The effects of PGMC and monolaurate (propylene glycol ester of LRA) on the growth of *M. furfur* were compared by culturing the fungus either in the presence of increasing concentrations of the fatty acid esters or at a single dose for different durations. These experiments showed that the esters behaved in a similar fashion as their parent fatty acids (Figures 3D,E) in both dose response (Figure 4C) and the time kill kinetics (Figure 4D). In both instances, while PGMC caused rapid fungal kill, monolaurate initially inhibited fungal growth but the effect saturated at higher doses or at longer duration using a dose of 0.2%.

### Combination of PGMC With Azole Antifungals Shows Increased Potency Against Resistant *C. albicans*

**FIGURE 5:**
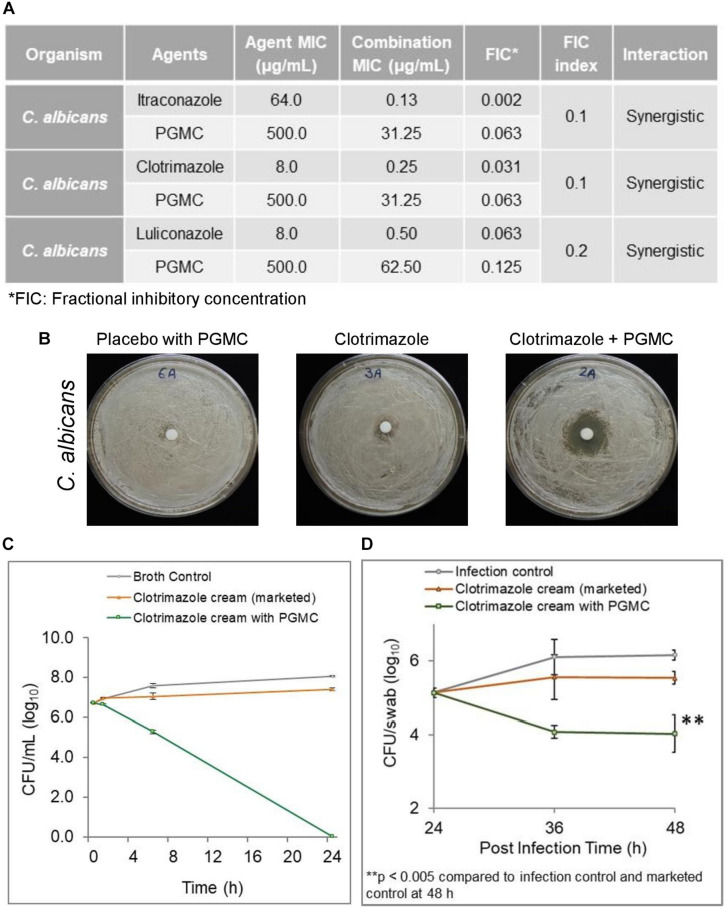
Efficacy of PGMC in combination with various antifungal agents against *Candida albicans*. **(A)** Combination effect of PGMC and antifungal agents against drug-resistant *Candida albicans* determined using checkerboard assays. **(B)** *In vitro* zone of inhibition assays using topical formulations containing no antifungal agent (placebo cream with PGMC), marketed formulation containing 1% clotrimazole and formulation containing 1% clotrimazole plus PGMC against *Candida albicans* **(C)** *In vitro* time kill kinetics of clotrimazole formulation containing PGMC versus marketed formulation of clotrimazole against *C. albicans* **(D)** Efficacy of clotrimazole (1%) formulation containing PGMC in a mouse model of cutaneous candidiasis. Fungal load was evaluated at designated time intervals during the efficacy experiment after treatment with antifungal formulations, clotrimazole (1%) topical cream containing PGMC/marketed cream of 1% clotrimazole. The infection control did not receive any treatment. Treatment time points were 24, 36, and 48 h post infection. The data is represented as mean ± SD (*n* = 4). Statistical analysis was performed using Student’s *t*-test (^∗∗^*p* < 0.005).

Since membrane disruption involves a non-specific mode of action, we asked whether PGMC potentiates commonly prescribed antifungal agents such as the azole drugs. Checkerboard assays showed PGMC to be synergistic with several azoles against an azole resistant *C. albicans* giving FIC indices as low as 0.1–0.2 (Figure 5A). The zones of inhibition were significantly increased when the resistant cultures were treated with a formulation containing a combination of PGMC and clotrimazole (1%) compared to formulations containing 1% clotrimazole alone (Figure 5B). Additionally, the 1% clotrimazole formulation containing PGMC was also superior to the marketed 1% clotrimazole formulation in an *in vitro* time kill assay (Figure 5C). These *in vitro* findings were validated in a murine cutaneous candidiasis model (Figure 5D). The clotrimazole formulation containing PGMC was significantly superior in reducing the load of azole resistant *C. albicans* (>2 log) while the marketed formulation barely reduced the fungal load. Interestingly, the early onset of antifungal activity of PGMC observed in the *in vitro* assays was also reflected in this *in vivo* model of fungal infection.

### Combination of PGMC With Known Antifungal Agents Shows Enhanced Potency Against Drug-Resistant Dermatophytes

**FIGURE 6:**
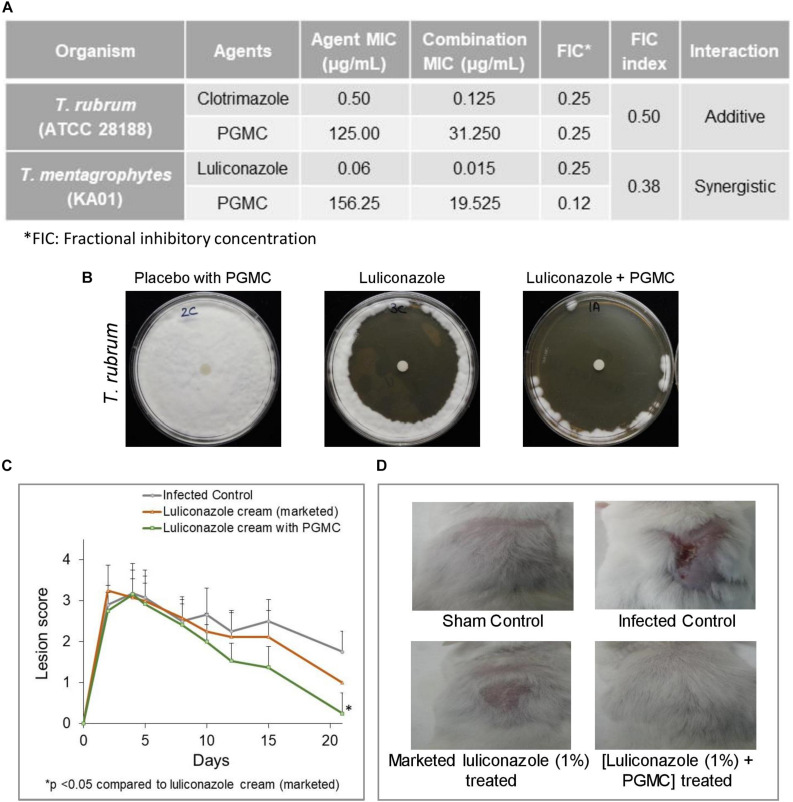
Efficacy of PGMC in combination with various antifungal agents against *Trichophyton* spp. **(A)** Combination effect of PGMC and antifungal agents against drug susceptible *Trichophyton rubrum* (ATCC 28188) and resistant *Trichophyton mentagrophytes var interdigitale* (KA01). determined through checkerboard assays. **(B)** Representative images of *in vitro* zone of inhibition assays using topical formulations containing no antifungal agent (placebo cream with PGMC), marketed formulation containing 1% luliconazole and formulation containing 1% luliconazole plus PGMC against *Trichophyton rubrum* **(C)** Efficacy of luliconazole (1%) formulations in a murine tinea infection model. Left panel showing effect of luliconazole formulation containing PGMC on skin lesion in a *Trichophyton mentagrophytes* (ATCC 24953) infected mouse model. Infection was initiated on Day 0 and treatment was started on Day 5 post infection and continued till Day 14. The data is represented as mean ± SD (*n* = 4). Statistical analysis was performed using Student’s *t*-test (^∗^*p* < 0.05). Topical luliconazole formulation containing PGMC showed significant (*p* < 0.05) antifungal effect relative to marketed formulation of 1% luliconazole (21 days post infection). **(D)** Representative pictures of lesions on mouse skin on day 21 in the tinea infection model.

To check the broad range activity of PGMC, it was tested against drug sensitive and resistant *Trichophyton* spp. using checkerboard assays. We found that PGMC has antifungal potentiating activity against *Trichophyton* spp., in most cases the combination leading to additive activity while in one case resulting in synergistic activity (Figure 6A). Next, we compared a marketed formulation of 1% luliconazole against formulations containing 1% luliconazole and PGMC in zone of inhibition (ZOI) assays. Although the marketed luliconazole formulation performed well in the ZOI assays, we consistently observed larger zones of clearance on the plates with the formulation containing 1% luliconazole and PGMC (Figure 6B). Finally, a topical formulation containing 1% luliconazole and PGMC was tested in a 21 days long tinea infection model in mouse (Figures 6C,D). The formulation containing 1% luliconazole plus PGMC showed a significant (*p* < 0.05) antifungal effect relative to marketed formulation of 1% luliconazole (21-days post infection) in terms of lesion scores and healing. When compared to the lesion scores for the marketed luliconazole cream treated animals, the PGMC containing 1% luliconazole formulation consistently gave better outcomes. Although fungal load was not evaluated in this study, faster healing of lesions in the animals treated with the PGMC containing luliconazole formulation suggested early onset of fungal clearance and perhaps barrier repair benefits of the formulation. Preliminary histological analyses (H&E staining) of day 21 skin samples from different arms revealed that skin from infected control (animal that did not receive any topical treatment) had features of lesions with hyperkeratosis, acanthosis, rete peg prolongation and presence of keratin cysts (KC) in the dermis (Supplementary Figure 3). In addition to the features seen in infection control, significant amount of ulceration with acanthosis at the edges (shown by black arrow) of the ulcerated area were seen in skin treated with marketed formulation of 1% luliconazole. Interestingly, murine skin treated with formulation containing combination of luliconazole (1%) with PGMC (8%) showed absence of such histopathological findings. These findings nicely corroborate with the healing of lesions in the mice that received the PGMC containing formulations wherein hair growth was also observed as a sign of complete healing (Figure 6D).

## Discussion

The global incidence of fungal infections is on the rise with an alarming increase in mortality, especially in the immune compromised population (Brown et al., 2012). To understand the real-life application of our technology we have focused on recalcitrant skin infections. This has two advantages. Firstly, localized application of antifungal formulations enables evaluation of the benefit of combining PGMC with azoles in a therapeutic dosage form applied directly at the site of infection, bypassing the systemic route. Secondly, commensal fungi like certain *Candida* spp. that commonly reside on healthy skin (Findley et al., 2013), often increase in abundance and become pathogenic during host immunodeficiencies (Oh et al., 2013). Among the known *Candida* species, *C. albicans* is most commonly responsible for symptomatic skin infections (Havlickova et al., 2008). The global trends of increasing azole resistance in various pathogenic *Candida* spp. is concerning as azoles are the class of antifungals used most frequently to treat *Candida* infections (Whaley et al., 2017).

The basidiomycete fungus, *Malassezia*, is a common commensal on the human skin, which can undergo transition to a pathogenic form under favorable conditions (Castellá et al., 2014). *Malassezia* is associated with folliculitis (Akaza et al., 2009), some forms of atopic dermatitis and psoriasis (Jagielski et al., 2014), and inflammatory dermatoses, such as dandruff and seborrheic dermatitis (Gupta et al., 2004). Indeed, the latter two skin conditions are extremely common, affecting more than 50% of adults, and are associated with a high socioeconomic impact and affect quality of life (Hay and Graham-Brown, 1997; Bickers et al., 2006). These *Malassezia*-associated diseases are often difficult to treat and require prolonged use of antifungal agents that may not be clinically optimal (Turner et al., 2012). Hence, there is a need to identify new therapeutic strategies that are effective in the treatment of *Malassezia*-associated skin disorders.

The worldwide prevalence of skin fungal infections is about 20% (El-Gohary et al., 2014), and the last decade has seen widespread increase in chronic and recurrent dermatophyte infections (Havlickova et al., 2008), especially in tropical countries like India (Dogra and Uprety, 2016). There is also increased reporting of resistance to common antifungal drugs used to treat dermatophytosis in recent years (Mukherjee et al., 2003; Yamada et al., 2017; Rudramurthy et al., 2018; Singh et al., 2018; Suzuki et al., 2018). This results in persistent infections that often cause considerable morbidity and lead to social, financial and emotional distress for patients (Dogra and Uprety, 2016). In addition to antifungal resistance, uncontrolled use of topical antifungal-corticosteroid combination therapy in some countries has led to extensive and difficult-to-treat dermatophytosis (Verma, 2018). The increasing trends of ineffective topical therapies of skin fungal infections are of concern as topical therapies limit the risk of systemic side effects and provide treatment directly targeted to the site of infection (Havlickova and Friedrich, 2008). However, potentiation of some of the antifungal compounds with antifungal fatty acids might prolong their efficacious topical use. In this work, we explored the use of a fatty acid (CAP) and preferably its GRAS derivative (PGMC) to potentiate antifungal agents via stepwise investigation of its *in silico* mechanism of action to *in vivo* efficacy in disease models.

The antifungal effects of CAP via a possible action of membrane penetration and disruption was investigated using MD simulations. We adopted the MARTINI CG model (Marrink et al., 2007; Marrink and Tieleman, 2013) for studying large length- and time-scale phenomenon which are difficult to capture using more detailed all-atomistic simulation models. The simulations suggested a distinct membrane penetration action of CAP, that was not displayed by LRA, a known antimicrobial fatty acid (Kabara et al., 1972). Measurement of multiple parameters (energy components of the system, packing of the bilayer, and displacement of phospholipids in the bilayer) over time demonstrated favorable interaction of CAP with the bilayer, allowing its entry and eventual penetration of the membrane leading to altered membrane dynamics (Figure 1). Interactions of surfactants with lipid membranes have been investigated in the past. Vermaas et al. (2017) studied membrane permeation by alkyl fatty acids compared to alcohols, aldehydes, and alkyl chains, and their extraction into a dodecane layer using atomistic MD simulations. They observed that transfer of fatty acid and alcohol between the membrane leaflet is slower compared to the other compounds. Groot and Rabone used mesoscale dissipative particle dynamics simulations to study the interaction of alcohol ethoxylates, a class of non-ionic surfactants, with phospholipid membranes and demonstrated formation of holes in the lipid bilayer (Groot and Rabone, 2001). In contrast, we explored the penetration of fatty acids into lipid membrane from water layer using coarse grained MD simulations. We show that penetration of the CAP molecules into phospholipid bilayer is guided by the balance of hydrophobic interactions among the fatty acid molecules and those between fatty acid self-aggregates and phospholipid. On the other hand, the LRA-LRA interaction energy favors the retention of LRA molecules in their self-aggregated clusters. Bilayer penetration by CAP molecules was found to be concentration dependent (Figure 2). 122–272 CAP molecules interacted minimally with the POPC bilayer. Bilayer penetration was apparent with 522 CAP molecules but did not cause membrane perturbation, while 1000 CAP molecules caused distinct membrane disorder as revealed by the steep rise in diffusion constant in this system (Figure 2F).

The *in silico* findings corroborated with *in vitro* findings, where, in the latter systems CAP not only displayed membrane penetration but caused extensive perturbation of the bilayer eventually leading to bilayer disruption. Fluorescence anisotropy measurements showed CAP caused an abrupt change in membrane fluidity in *M. furfur* cells while LRA caused minimal changes. TEM visualization revealed destruction of all membrane structures in *Malassezia* upon short duration exposure to CAP whereas LRA had no effect. Short/medium chain fatty acids and their mono-glycerides have been previously studied for their microbial growth modulatory properties (Kabara et al., 1972; Batovska et al., 2009). For example, LRA was reported to be inhibitory in a variety of microbes (Kabara et al., 1972) but our *in vitro* data suggests that it is not effective for *M. furfur* growth inhibition. LRA is a saturated fatty acid with four extra carbons compared to CAP and exerts a distinct effect on *M. furfur* cells, indicating chain length could contribute to specificity in antimicrobial action of fatty acids through differential membrane perturbation in different fungi. The membrane damaging effects of CAP translated to dose-dependent *in vitro* growth inhibition and cell death. An ester of CAP (PGMC) displays similar fungal kill properties as the parent fatty acid and achieved equivalent *in vitro* antifungal potency as CAP (Figure 4). This was important as PGMC is already approved by the USFDA as a GRAS excipient, which means its use could be translated rapidly to the clinic. A topical formulation combining PGMC with piroctone olamine was found to exert an early onset of therapeutic benefits and alleviation of symptoms in patients with moderate dandruff (Bhattacharyya et al., 2017).

Moving forward with the observed benefits in anti-dandruff therapy, we explored the possible advantages of including PGMC in topical antifungal formulations clinically relevant for the treatment of *Candida* or *Trichophyton*-associated skin infections. We found that PGMC combined with various azoles shows synergistic inhibitory activity against *C. albicans* (Figure 5) and mostly additive and in one case synergistic inhibition when tested against various *Trichophyton* spp. (Figure 6). This combination effect was also apparent in antifungal formulations containing PGMC which demonstrated superior *in vitro* fungal kill compared to marketed azole antifungal formulations. The PGMC containing formulations clearly outperformed their marketed counterparts in efficacy models of *in vivo* cutaneous fungal infections (Figures 5, 6). The two animal models used in this study are considered standard and reliable for study of the pathogenesis and treatment of skin fungal infections (Capilla et al., 2007; Shimamura et al., 2012). The early onset of action and superior healing of lesions by the PGMC containing formulations have promise in topical therapy of dermatophytosis as this may encourage patient compliance and thereby reduce opportunity for relapse.

Aspects of this study may directly impact the treatment of resistant and difficult-to-treat fungal infections. The *in silico* platform used in this study may help search for novel antifungal activities of known molecules. The identification of the GRAS material PGMC as an antifungal agent that potentiates different antifungal drugs indicates the possibility of developing new delivery technologies that may increase the efficacy of existing antifungal therapies. Alternatively, as the derivatization of CAP did not impact its antifungal activity, novel molecules created by conjugating the CAP backbone with existing antifungal agents might generate new antifungals that exhibit bifunctional mechanisms of action. The reproducible results obtained using the well-accepted and standard *in vivo* infection models provide confidence to explore the reach of this platform in more challenging cases of fungal infections. The current study has expanded our options for dealing with the emergence of drug-resistant fungal diseases.

## Data Availability Statement

All datasets generated for this study are included in the article/Supplementary Material.

## Ethics Statement

The animal studies were reviewed and approved by the institutional animal ethics committee (Protocol Nos. IAEC/01/2016/008 and IAEC/02/2016/018) at TheraIndx Lifesciences Pvt. Ltd. (Bangalore, India).

## Author Contributions

ShG and SS were responsible for all the simulation data with the model membrane. AB, MS, and ShG designed and supervised the *in vitro* and *in vivo* studies. SuG supervised the preparation of formulations. HS and RSP performed the *in vitro* assays. AB and MS analyzed the data. AB, MS, and ShG drafted the manuscript. KS provided clinical perspectives. KS and SS reviewed the manuscript with expert opinion. All authors contributed to the article and approved the submitted version.

## Conflict of Interest

SS and ShG hold equity in Vyome Therapeutics Ltd. SS was a consultant for Vyome Therapeutics Ltd. KS was an academic collaborator of Vyome. AB, ShG, MS, HS, and SuG employees of Vyome Therapeutics Ltd. RSP was a former employee of Vyome.

## Footnotes

1 https://www.cdc.gov/fungal/antifungal-resistance.html

2 http://www.who.int/mediacentre/factsheets/fs194/en/

3 http://www.cgmartini.nl
