# Supplementary material for: Mechanistic Insight Into the Antifungal Effects of a Fatty Acid Derivative Against Drug-Resistant Fungal Infections
Source: Front Microbiol. 2020 Sep 8;11:2116. doi: 10.3389/fmicb.2020.02116 (PMC7505954; doi:10.3389/fmicb.2020.02116)
Supplement: Supplementary file 1 [file Data_Sheet_1.docx]

**Mechanistic insight into the antifungal effects of a**

**fatty acid derivative against drug-resistant fungal**

**infections.**

Anamika Bhattacharyya^1↑^, Mau Sinha^1↑^, Himanshi Singh^1^, Ranjeet Singh Patel^1^, Sumana Ghosh^1^, Kabir Sardana^3^, Shamik Ghosh^1,2^*, Shiladitya Sengupta^4^*

^1^Vyome Therapeutics Ltd, Delhi, India.

^2^India Innovation Research Center, Delhi, India.

^3^Department of Dermatology, Atal Bihari Vajpayee Institute of Medical Sciences and Dr Ram Manohar Lohia Hospital, New Delhi, India.

^4^Department of Medicine, Division of Engineering in Medicine, Brigham and Women’s Hospital, Harvard Medical School, Boston, MA 02115, USA.

↑Contributed equally.

***Correspondence**:

Dr. Shamik Ghosh

[shamik.ghosh@vyometx.com](mailto:shamik.ghosh@vyometx.com)

Dr. Shiladitya Sengupta

[ssengupta2@bwh.harvard.edu](mailto:ssengupta2@bwh.harvard.edu)

**Supplementary Figure 1.** Adaptation of the MARTINI Coarse Grain (CG) model. Representations of MARTINI CG bead types for POPC and caprylic acid (CAP) molecules are shown. Bead names and the corresponding types are indicated.

**Supplementary Figure 2.** Propylene glycol monocaprylate (PGMC) causes dramatic changes in *C. albicans* even at short duration exposures. (**A**) Scanning electron micrographs of *Candida albicans* (ATCC 22972) after a 3 h treatment with vehicle (top, left) and PGMC (top, right), luliconazole (bottom, left) or a combination of luliconazole and PGMC (bottom, right).


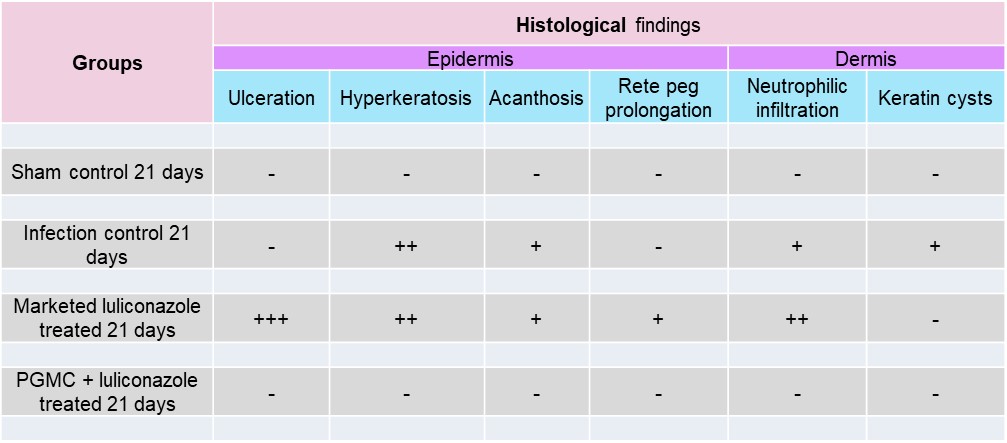


**Supplementary Figure 3.** Histological findings from skin samples of murine tinea model. Haematoxylin and eosin stained skin samples from day 21 mice from different arms of the study were analysed for histo-pathological findings as scored in the table above. Representative images for sample from each arm are shown below the table. Keratin cysts (KC) are indicated in the image for infected control and black arrows indicate ulceration and acanthosis in the image for marketed luliconazole treated arm.

**Supplementary Movie LRA-1000**. Movie depicting changes in POPC bilayer in the presence of 1000 LRA molecules.

**Supplementary Movie CAP-1000**. Movie depicting changes in POPC bilayer in the presence of 1000 CAP molecules.

**Supplementary Movie CAP-522**. Movie depicting changes in POPC bilayer in the presence of 522 CAP molecules.

**Supplementary Movie CAP-272**. Movie depicting changes in POPC bilayer in the presence of 272 CAP molecules.

**Supplementary Movie CAP-122**. Movie depicting changes in POPC bilayer in the presence of 122 CAP molecules.
